# Weight changes in esketamine nasal spray and quetiapine extended-release treated patients with treatment resistant depression: Results from ESCAPE-TRD study

**DOI:** 10.1192/j.eurpsy.2024.626

**Published:** 2024-08-27

**Authors:** A. Reif, A. Fagiolini, E. Buntinx, H. Ruggeri, Y. Godinov, J. Buyze, S. Mulhern-Haughey, I. Bitter

**Affiliations:** ^1^Goethe University Frankfurt, University Hospital, Department of Psychiatry, Psychosomatic Medicine and Psychotherapy, Frankfurt; ^2^Fraunhofer Institute for Translational Medicine and Pharmacology ITMP, Theodor-Stern-Kai 7, 60596, Frankfurt am Main, Germany; ^3^Department of Molecular Medicine, University of Siena School of Medicine, Siena, Italy; ^4^Medical Center Anima, Alken, Belgium; ^5^CEN (Centro especializado en Neurociencias), Córdoba, Argentina; ^6^Janssen EMEA, Sofia, Bulgaria; ^7^Janssen Pharmaceutica NV, Beerse, Belgium; ^8^Janssen EMEA, Dublin, Ireland; ^9^Department of Psychiatry and Psychotherapy, Semmelweis University, Budapest, Hungary

## Abstract

**Introduction:**

In ESCAPE-TRD, esketamine nasal spray (ESK-NS) significantly increased the probability of remission at Week (Wk)8 and being relapse‑free through Wk32 after remission at Wk8 versus (vs) quetiapine extended-release (QTP-XR), in patients (pts) with treatment resistant depression (TRD). Safety data were consistent with established profiles of each treatment, with no new safety signals identified (Reif *et al.* DGPPN 2022; P-01-04).

**Objectives:**

To explore weight changes and their impact on treatment discontinuation in ESCAPE-TRD.

**Methods:**

ESCAPE‑TRD (NCT04338321) was a randomised, open-label, rater-blinded, phase IIIb trial comparing efficacy and safety of ESK-NS vs QTP-XR in pts with TRD. Safety analyses were conducted on pts who received ≥1 dose of study treatment. Treatment-emergent adverse events (TEAEs) were defined as occurring at or after the first dose of study treatment and within 14 days/30 days (non-serious/serious) of the last dose. A ≥7% increase/decrease in weight from screening was considered for evaluation as a TEAE. Weights were measured and are reported as observed, with no missing data imputation.

**Results:**

336 and 340 pts were randomised to ESK-NS and QTP-XR; 334 and 336 were included in the safety population. Over the 32-week study, a TEAE of weight increase was reported in fewer pts treated with ESK-NS than QTP-XR (9 [2.7%] vs 42 [12.5%]), leading to treatment discontinuation in 0 vs 6 (1.8%) pts, respectively. Incidences of weight increase TEAEs were balanced across pts categorised as normal, overweight or obsese by baseline body mass index (BMI; **Figure**). A weight decrease TEAE was reported in 7 pts (2.1%) in the ESK-NS arm vs 0 pts in the QTP-XR arm. Mean (standard deviation [SD]) weight at baseline was 76.4 (16.2) kg (ESK-NS; n=334) vs 79.1 (16.9) kg (QTP-XR; n=336). At Wk32, mean weight was maintained (76.5 [16.3] kg) in ESK-NS treated pts (n=249; mean [SD] change from baseline: 0.1 [4.0] kg) and increased (80.7 [15.6] kg) in QTP-XR treated pts (n=203; mean [SD] change from baseline: 2.5 [5.1] kg).

**Image:**

**Figure:**
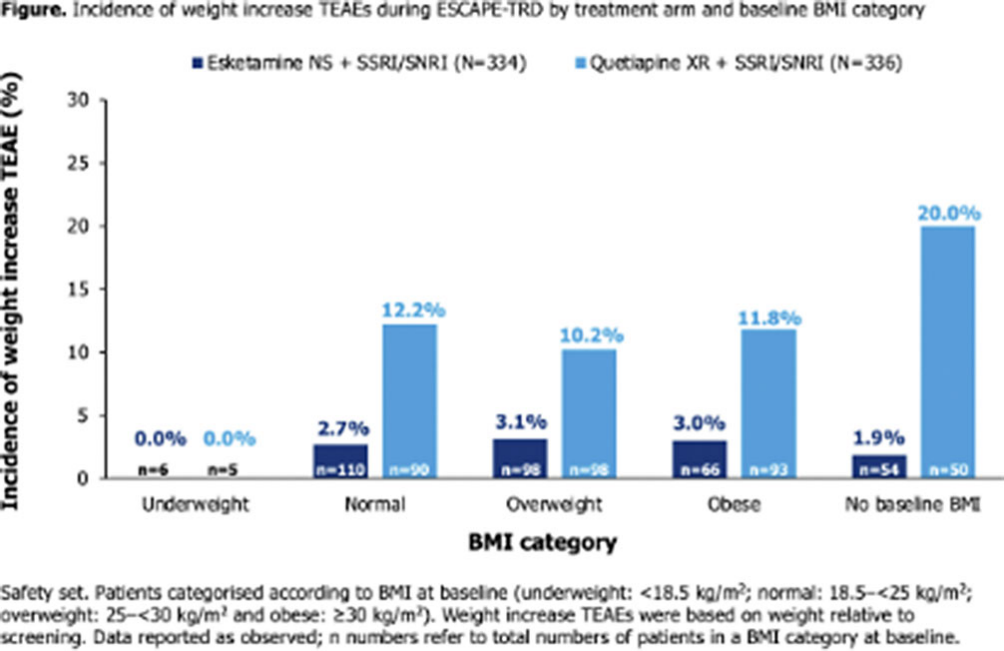

**Conclusions:**

Increase in weight was uncommon with ESK-NS; weight increases were more common with QTP-XR and resulted in more treatment discontinuations. Weight increase was independent from baseline BMI.

**Acknowledgements:**

We thank the patients who participated. Funding: Janssen, medical writing: Costello Medical, UK

**Disclosure of Interest:**

None Declared

